# Elective egg freezing: what is the vision of women around the globe?

**DOI:** 10.2144/fsoa-2019-0068

**Published:** 2020-03-31

**Authors:** Susan Nasab, Lindsey Ulin, Chikara Nkele, Jaimin Shah, Mazen E Abdallah, Baha M Sibai

**Affiliations:** 1 Department of Obstetrics & Gynecology, McGovern Medical School, The University of Texas Health Science Center at Houston, Houston, TX 77030, USA; 2 Houston Fertility Institute (HFI), TX 77030, USA

**Keywords:** elective egg freezing, fertility preservation, oocyte cryopreservation, social egg freezing

## Abstract

**Aim:**

The interest in oocyte cryopreservation (OC) for nonmedically indicated reasons is increasing. Knowing women’s beliefs and knowledge from various geographic regions could help providers to understand the similarities and differences that could facilitate proper counseling.

**Materials & methods:**

Articles about social egg freezing published over the past 18 years were extracted from the literature.

**Results:**

We demonstrated that there are common rationales toward OC among women in the USA and other countries. The ultimate goal was to prolong fertility. The most commonly reported reasons were aging, lack of partner, career and financial status.

**Conclusion:**

The beliefs and rationales toward elective OC among women in the USA and other countries are consistent.

## Lay abstract

The interest in oocyte cryopreservation for nonmedical reasons, known as elective egg freezing, is increasing, owing to several personal and social factors. The understanding of the attitudes and beliefs of women from a variety of geographies could aid in the provision of proper counseling. This review examined prior literature and demonstrated that there are common rationales for oocyte cryopreservation, including aging, lack of partner, career, financial status and not being ready to have a child. It highlighted that there is a necessity for further education and awareness.

In the USA, the birth rate for women 40–44 years of age has risen over the last three decades. The 2015’s rate for this age group is the highest since 1966 (11 births per 1000 women) [1]. Based on birth data released by CDC, birth rates have declined for women in their 20s, but increased for women in their 30s and early 40s [1].

This trend, which is not only identified in the USA, has been affecting childbearing decisions among women across the globe. Multiple nonmedical or social reasons have influenced family planning among reproductive aged women [2].

Elective egg freezing, also called social egg freezing, is defined as oocyte cryopreservation (OC) for potential future use by women who are choosing to delay childbearing for personal reasons, such as career pursuits, advancement in education, lack of a partner or financial stability. The first successful human OC and fertilization was reported in 1986 by C Chen, during which he achieved a successful twin pregnancy [3]. Cryopreservation for medical reasons, such as cancer, is already considered as an established method, but it is a fairly new concept for social reasons [4–7]. In 2008, the American Society of Reproductive Medicine announced OC as experimental and suggested that more research and investigations are needed over a longer period [8]. Since then multiple investigators have studied OC in more depth and numerous papers have been published. In 2010, Cobo *et al.* conducted a randomized controlled trial which examined pregnancy rates of 600 young women who received either fresh or vitrified donor oocytes, and interestingly found no difference in pregnancy rates [9]. Finally, in 2013, the American Society of Reproductive Medicine announced that elective oocyte preservation is no longer considered experimental [10]. However, due to limited data on emotional affects, safety, efficacy, ethics and cost–effectiveness, they did not recommend elective OC, but continued to support cryopreservation for medical reasons, such as cancer treatment.

Prior studies have reported personal and social reasons for pursuing elective egg freezing [11–18]. In the USA, the most common indications for elective OC include aging, lack of a partner and financial constraints [2,19].

However, moving from medical OC to social egg freezing, reflections concerning the social debate raised by this practice focused on marketing, interpretation of the impact and meaning of such technology and needs to be addressed. Can social egg freezing lead to improvement in a woman’s quality of life and a decrease in fertility anxieties? This important question has been discussed in previous literature and by different societies [20]. The USA is considered a country with residents from all over the world, with a diverse culture and background. Knowing women’s attitudes, beliefs and knowledge from a variety of geographic regions can help providers to understand the similarities and differences, which could facilitate proper approach and counseling. Hence, we were interested in identifying the beliefs and attitudes toward social egg freezing around the world.

## Materials & methods

### Inclusion criteria

Primary articles that studied women interested in elective egg freezing were initially selected. Only studies designed as a survey or interview and published during 2000–2018 were included. The outcomes were to evaluate attitudes and beliefs toward elective egg freezing. In general, the responders who believed OC to be a potential fertility preservation option were reported as ‘Supporters’ and the responders who were in disagreement the option were considered as ‘Nonsupporters’. Only articles written in English were included.

### Review of literature & study selection

Pubmed, Medline and Cochrane were searched. The following medical subject headings, major topics or terms were used for the literature search: elective egg freezing, social egg freezing, oocyte cryopreservation and nonmedically indicated egg freezing. Two independent reviewers reviewed all primary selected articles. Full manuscripts of the relevant articles were reviewed carefully, and all nonrelevant papers, which were selected by electronic search, were excluded. The studies, which were designed as a survey or interview, were included in the study.

Demographic characteristics of the survey responders such as age, relationship status, educational level, as well as their positive/supportive versus negative/nonsupportive beliefs toward social egg freezing were reported. The studies are ordered chronologically in Table 1, based on the period of when the study was performed, not based on the year of publication [2,11–17,19,21–34].

**Table 1.** Study characteristics.

Study (year)^†^	Country	Type of survey	Numbers (F/M)^‡^	Age range (mean)	Population	Educational level	Marital status	Beliefs	Ref.
Lampic *et al.* (2004)	Sweden	Postal	401 (222/179)	(24)	Uppsala University students	Undergraduate	50% in relationship	N/A	[30]
Vallejo *et al.* (2005–2006)	USA	Chart review	20	(38.6)	Women who underwent elective OC cycle at Reproductive Medicine Associations of New York	100% bachelor, 75% masters or professional degree	Single	– Prolong fertility	[19]
Hodes-Wertz *et al.* (2005–2011)	USA	Online and postal	183	(38)	Women who underwent OC at fertility center at New York Langone Medical Center	N/A	Never married	Reasons for not pursuing childbearing earlier: – Aging – Lack of partner – Professional/financial reasons – Too large of commitment	[20]
Gorthi *et al.* (2009)	UK	Paper questionnaire	200	18–30	Medical students vs nonmedical students at Leeds University	University	63% medical students single, 25.8% nonmedical students single	Reasons to postpone family: – Career – Financial stability – Marriage	[31]
Liu *et al*. (2010)	Canada	Online	20	30–42	Medical directors of Canadian ART Clinics	N/A	N/A	– Prolong fertility	[15]
Stoop at al. (2010)	Belgium	Online	1024	21–40 (28.5)	Women in national database	Medium–high	Married or cohabitating	– No effect on future fertility – Health and safety of children – More financial reimbursement – More guarantee for success – Treatment less complex – If no children yet	[13]
Bavan *et al.* (2011)	USA	Online	328	17–31 (22)	Female students in various universities in California	University	51% in relationship	N/A	[32]
Brezis *et al.* (2011)	Israel	Online, interview, telephone	840		– ART unit directors – Bioethics experts – Medical students – Members of general population	Graduate	N/A	N/A	[33]
Lallemant *et al.* (2012)	UK and Denmark	Online	973	18–68 (31)	General population	50% above high school	81% in relationship	1. Positive rationales: – Young age – Single status – Previous divorce – No children yet – Difficulty conceiving – Part-time job – Medical indication (cancer) 2. Negative rationales – Career reasons – To allow time to find right partner – Financial reasons	[9]
De Groot *et al.* (2012)	The Netherlands	In-person interview	20	(36)	Women on waiting list for oocyte banking at a Dutch University medical center	University > college > secondary vocational education	Single	1. Positive rationales – To prolong fertility, – Resulting children are healthy – Time to find partner 2. Negative rationales – Pain of follicle aspiration – Hormonal medication	[12]
Tan *et al.* (2012)	Singapore	Online	129	20–31	Female medical students	Graduate	N/A	– Consider OC if government subsidy – Career reasons – Current lack of partner, – Age >30	[24]
Wennberg *et al.* (2013)	Sweden	Postal	1661, (987, 674)	30–39	General population: – Stockholm (urban cohort) – Any part of Sweden (national cohort)	University > college > basic school	Married or cohabitating	1. Positive rationales – Medical reasons (94%) – Urban cohort more positive than national cohort – Social reasons (70%) – Single status	[10]
Yu *et al.* (2014)	USA	Online	239 (216, 23)	26–30	Ob/GYN resident physicians	MD	N/S	1. Positive rationales – Cancer diagnosis – Inform patients 2. Negative rationales – Career reasons – Young age – Not pressure patients into childbearing	[34]
Ter Keurst *et al.* (2014)	UK	Online	257	28–35 (31)	Childless women who wished to have children	University	In relationship	– Achieve parenthood – Having children at a later age – Fewer ethical concerns	[35]
Stoop *et al.* (2014)	Belgium	Telephone	138	(43)	Women seen at clinic for banking	N/A	50% in relationship	– Insurance against future infertility – More time to find partner – Prevent regret	[27]
Daniluk *et al.* (2015)	Canada	Online	500	18–38	Childless, presumed fertile women	College or university > high school > post-grad	No partner > married/ cohabitating	– Medical reasons (Cancer) – No partner – Self or partner not ready to have children	[11]
Lewis *et al.* (2015)	USA	Online	1064 (590, 474)	18–65	General population	Bachelor degree > graduate degree > some college	Married > never married	1. Positive rationales – Cancer diagnosis – Career plans – Current lack of partner – Insufficient funds for child rearing – Higher income – Atheism or agnosticism – No children yet 2. Negative rationales – Unethical – Creation of older parents unable to care for their children – Older age	[18]
Baldwin *et al.* (2015)	UK	Online/referrals	23	36–39 (36.7)	Women who underwent elective OC	University, 65% postgraduate or professional qualifications	87% not in relationship	– Prolong fertility	
Kilic *et al.* (2016)	Turkey	Interview	21	Median age 40	Women who underwent/in the process of elective OC	University degrees in all except one who was high school graduate)	All unmarried	– Positive influence on the future relationship – Preserve fertility – Boost self-esteem and self worth – Concern to be rejected by men due to lost fertility	
Kim *et al.* (2016–17)	South Korea	Paper questionnaire	91	(37.1)	Women who underwent elective OC	Not reported	85% Single	– Lack of partner – Professional reasons – Insurance against future fertility	
Inhorn *et al.* (2014–16)	USA Israel	Interview	114	25–>40 (36)	Women who underwent elective OC	72% graduate degree	78% Single	– Women have higher expectations – Career – Lack of right partner	
Esfandiari *et al.* (2018)	USA	Online	113 (103/10)	26–>35	Ob/GYN residents and fellows	100% graduate degree	58% married, 23% single	– Lack of partner	

† The year of the study when the survey was performed is reported.

‡ The study participants are female, except the ones in parenthesis, which represent female and male responders.

ART: Assisted reproductive technology; GYN: Gynecology; MD: Medical degree; Ob: Obstetrics; OC: Oocyte cryopreservation; N/A: Not applicable.

## Results

A total of 1135 citations were retrieved electronically based on the search strategy, of which 1078 were excluded through review of title or abstract. Full manuscripts of 57 published articles about OC for nonmedically indicated reasons were reviewed in detail. Subsequently 22 original studies, which were designed as a survey or interview, were included in our review. Out of those 22 studies, 13 (59%) were designed as online surveys, and the rest were designed as postal or paper questionnaires, telephonic surveys or in-person interview. A total of 17 (77%) studies included females only. Moreover, 10 out of 22 were performed in European countries, 7 in the USA, 2 in Canada, 2 in the Middle East and 2 in East Asia (the different characteristic and findings of reviewed studies are summarized in Table 1).

### Positive rationales

We commonly found that the rationales for electing to cryopreserve oocytes centered on goals that women wanted to accomplish prior to having children. Some of the goals included accomplishments such as finding a partner, achieving more financial stability and career-related goals.

In general, there was significant support toward elective egg freezing by American and Non-American populations, mainly by single or childless women, or women with more financial and career stability. Some of the common trends seen among women in Belgium, Denmark, Sweden, USA and UK in support of elective cryopreservation, included allowing more time for financial stability, more time to find a partner and success rates of procedures (Table 1). Women in the Netherlands who were considering oocyte banking stated that their wish to share parenthood with a future partner overruled their concern for possible complications from banking [14]. In a cross-sectional study of female medical students in Singapore, 46.5% of them cited finding a partner as a reason for OC [26]. A total of 66% of respondents from an electronic survey in Canada supported cryopreservation for young women [13]. From this same group of supporters the top four factors that influenced their decision were financial costs (85.6%), health risks to themselves (86.4%) or their offspring (87.8%) and success rates (82%) [13]. In a cross-sectional study of 1064 women in the USA, it was found that people supported elective OC based on the following criteria: delayed childbearing for career advancement (72%), current lack of a partner (63%) and insufficient funds for child rearing (58%) [2].

### Negative rationales

Although throughout the studies there was a lot of support for OC, there were some individuals who expressed dissent. Some of the rationales for their dissent were lack of evidence about effect on future fertility, finding OC unethical, complexity of treatment and creation of older parents who may not be able to care for their children. One study in the USA reported that an older age of 45–65 years was associated with lower support for OC [2]. In a study of women at a Dutch medical center on a waiting list for OC, they expressed their fear of pain from follicle aspiration, and emotional imbalance from hormones as reasons for being hesitant to move forward with the procedure [14].

## Discussion

Based on our review, overall women’s rationales toward OC in the USA echoed the same feelings of many women across the world in places such as the UK, Denmark, Belgium, Sweden, Singapore and the Netherlands.

This study has several strengths and limitations. A limitation of the study was that all types of surveys were included in the study, including online, telephonic, mail and so on. It would be ideal to have face-to-face interviews, or at least all the surveys designed in the same style. However, per our search, there are not many surveys regarding elective egg freezing around the world, and hence we included all available ones.

What is more, the difference in survey questions and their wording/phrasing (open ended vs straightforward questions) can affect the response of survey takers.

This review is strengthened by its heterogeneity. Original studies were performed in different geographic locations, enrolling different populations and ethnicities, with different personal, religious and ethical beliefs, and this increases the generalizability. Given the fact that the population in the USA is also very diverse, physicians can benefit from this information in terms of understanding their patient population, regardless of their background.

## Why is knowing patient beliefs & attitude important?

### Proper counseling

For physicians to select the appropriate patient population, to use reasonable and effective strategies and to counsel about elective egg freezing, it is important to consider the demographics, understandings, beliefs and concerns of the people who are interested. Overall, the most common demographics of people interested in elective OC were single, divorced or widowed, desiring future fertility, childless, having little ethical conflict and having medium to higher incomes. The average age of interested women, or women who had already undergone OC, was around 35. In an oocyte banking center in the Netherlands, the average age of women on their waiting list was 35.8 years [14]. In another center in New York, USA, more than 85% of women who had undergone cryopreservation were older than 35 years of age [19]. In a study conducted in Canada, which surveyed 16 of the 28 assisted reproductive technology clinics, authors found that all clinics offered cryopreservation as an option to women at 35–36 years, but the consensus varied among clinics for ages younger or older than this. The data show the importance of proper counseling of patients who are OC candidates and at an appropriate age [35], and their education about the success rate at each age group [10,36,37].

### Appropriate knowledge & awareness

The other important issue is the overall knowledge of women about fertility reserve and fertility preservation options. More importantly, do they have accurate information about fertility preservation success rate and outcomes? Lack of knowledge about female age-related fertility decline has been mentioned in previous studies [38]. In one of the online surveys of childless women in Canada, it found that women averaged 33% when questioned about infertility and treatment [13]. A survey of 129 Singaporean medical students surveyed via an online questionnaire found that only 36.4% of them had ever heard of social egg freezing [26]. In another study, 70% of surveyed women considered OC as a fertility preservation option, but after receiving more information about the procedure the proportion of potential egg freezers decreased to 48.9% [12]. Of 1914 women surveyed in Belgium between ages 21 and 40, women were more likely to engage in social egg freezing if they were educated and informed about the risk of the procedure, its effect on their future fertility (75.2%), and the health of children conceived from preserved oocytes (70.9%) [29]. In a cross-sectional study of 500 childless women in Canada, only 14.8% of them rated their knowledge of cryopreservation as ‘very’ knowledgeable, whereas 53% of them rated their knowledge as ‘some’ [39]. When these same participants were asked 12 knowledge questions about cost, risk and viability of oocyte freezing, most women showed a high level of uncertainty with an average of 33% on the test [39]. 61% of women believed that physicians should provide information to childbearing women at their annual visits [39]. Moreover, in a recent study in Italy among young female students, 34.3% have heard about OC for social reasons. They were significantly less aware of age-related decline in fertility and the possibility of using social egg freezing compared with their counterparts in other western countries [40]. Lack of knowledge of fertility is expected to continue to be a problem for those who are choosing to delay child-bearing, unless we educate them on the available preservation options.

Finally, in a recent survey of women who underwent OC, performed by Greenwood *et al.* [41], those who perceived that they have had adequate counseling reported less regret. 80% reported that they had adequate information before the process and 69% perceived adequate emotional support. Those with higher expectation of future success with oocyte preservations had lower odds of regret.

We would like to emphasize the crucial role of physicians to provide informed consent and accurate information, to avoid emotional crises and decrease the rate of regret [36].

## Conclusion

Based on our literature review, elective OC gives an option to certain women to slow down the fertility clock and align it with the time when they find themselves stable enough and sufficiently mentally, emotionally or financially prepared to have a child. Overall, the beliefs and rationales toward elective OC among women in the USA and with other countries appear consistent. Many women in their mid 30s would consider this as an option if they desire to have children in the future, but have not yet accomplished the financial, career or partner goals that they desire. Factors that inhibit women who are interested from actually acting on their interest include lack of finances, ethical dilemmas, complexity of treatment, lack of knowledge about the efficacy, effect on future fertility and viability of oocytes.

In general, knowledge about fertility treatment is low across the globe. As our population of childbearing women continues to age and as cryopreservation techniques and cost continue to improve, we expect there will continue to be a growing demand for elective oocyte services. Offering proper counseling and targeting the appropriate population of women who are interested in cryopreservation would be highly recommended for infertility providers and counselors.

## Future perspective

We speculate that in the next decade elective OC could be considered an option for family planning. Face-to-face survey studies in the future will provide a better picture of the rationales and beliefs toward elective egg freezing. Educational sessions and awareness programs are needed to provide information about available fertility preservation options, which could potentially decrease the rate of regret.

**Executive summary**

- The interest in oocyte cryopreservation for nonmedical reasons, known as elective egg freezing, is increasing, secondary to several personal and social factors.
- Knowing women’s attitudes and beliefs coming from other geographic regions could help practitioners to provide proper counseling.
- Among most populations, the most common demographics of people interested in elective oocyte cryopreservation were single, divorced or widowed, desiring future fertility, childless, having little ethical conflict and having medium to higher incomes.
- Rationales for elective egg freezing were mainly centered on goals that women wanted to accomplish prior to having children such as finding a partner, achieving more financial stability and career-related goals.
- Overall, there is a lack of knowledge about female fertility reserve and fertility preservation options around the globe.
- Lack of knowledge about female fertility reserve necessitates further education and awareness.
- Offering proper counseling and targeting the appropriate population of women who are interested in cryopreservation would be highly recommended to the infertility providers and counselors.
